# Design of Hybrid Gels Based on Gellan-Cholesterol Derivative and P90G Liposomes for Drug Depot Applications

**DOI:** 10.3390/gels3020018

**Published:** 2017-05-08

**Authors:** Nicole Zoratto, Francesca Romana Palmieri, Claudia Cencetti, Elita Montanari, Chiara Di Meo, Maria Letizia Manca, Maria Manconi, Pietro Matricardi

**Affiliations:** 1 Department of Drug Chemistry and Technologies, Sapienza, University of Roma, P.le A. Moro 5, 00185 Roma, Italy; nicole.zoratto@uniroma1.it (N.Z.); palmieri.1247758@studenti.uniroma1.it (F.R.P.); claudia.cencetti@gmail.com (C.C.); elita.montanari@uniroma1.it (E.M.); chiara.dimeo@uniroma1.it (C.D.M.); 2 Department of Scienze della Vita e dell’Ambiente, University of Cagliari, via Ospedale 72, 09124 Cagliari, Italy; mlmanca@unica.it (M.L.M.); manconi@unica.it (M.M.)

**Keywords:** P90G liposomes, gellan–cholesterol, hydrogel, depot, drug delivery

## Abstract

Gels are extensively studied in the drug delivery field because of their potential benefits in therapeutics. Depot gel systems fall in this area, and the interest in their development has been focused on long-lasting, biocompatible, and resorbable delivery devices. The present work describes a new class of hybrid gels that stem from the interaction between liposomes based on P90G phospholipid and the cholesterol derivative of the polysaccharide gellan. The mechanical properties of these gels and the delivery profiles of the anti-inflammatory model drug diclofenac embedded in such systems confirmed the suitability of these hybrid gels as a good candidate for drug depot applications.

## 1. Introduction

Over the past few decades, liposomes have been extensively studied for drug delivery applications, thanks to their unique properties, such as biocompatibility, low immunogenicity, low toxicity, and high versatility [1,2]. Indeed, both hydrophilic and hydrophobic payloads can usually be entrapped within liposomes through several preparation processes. Moreover, these vesicles are able to change the in vivo distribution of entrapped drugs, improving their accumulation in the target site [3]. Unfortunately, drug release is affected by vesicle stability in the in vivo environment and by the exposure to serum proteins, which can facilitate a rapid drug leakage. Additionally, conventional liposomes suffer from a rapid in vivo clearance due to their uptake into the cells of the mononuclear phagocyte system, which reduces their in vivo biodistribution, and may cause liver and spleen toxicity [4]. 

Many different approaches have been used to overcome these limits; for instance, the coating of liposomes with hydrophilic and flexible polymers able to form soft coronae tenuously adsorbed or anchored on the lipid membrane should improve their half-life in blood circulation, simultaneously modulating the drug release and the efficacy of the nano-carrier [5,6]. In this respect, Tabbakhian et al. [7] evidenced the stronger interaction of hydrophobically-modified polymers (e.g., cholesterol-modified mannan) with the liposome bilayers with respect to that of the native polysaccharide, thus forming a coating that did not hamper the delivery ability of vesicles but rather flowed with them, improving their performances.

Sekine et al. [8] developed a cholesterol-modified pullulan (CHP) nanogel for liposome coating: hydrophobic forces allowed cholesterol to penetrate into the lipid bilayer of liposomes, forming stable coated nano-complexes. Additionally, CHP-liposomes were entrapped into a hydrogel in order to obtain a depot delivery system. Indeed, depot represents another useful strategy capable of modifying the release properties of liposomes [9]. In this respect, several hydrogel systems have been studied and developed to obtain drug-loaded liposomes entrapped into prefabricated polymer scaffolds [10] or into in situ gelling systems [11]. The polymer hydrogel network has a key role in retaining, protecting, and modulating vesicle release, but if not appropriately formulated, it can prevent the vesicle’s passage to the biological fluid, avoiding the system’s efficacy. Consequently, the choice of an appropriate polymer structure represents a fundamental parameter for the design of an efficient liposomal depot system.

Gellan gum (Ge) is an anionic microbial polysaccharide with a tetrasaccharide repeating unit ((1-3)-β-d-glucose, (1-4)-β-d-glucuronic acid, (1-4)-β-d-glucose, and (1-4)-α-l-rhamnose), secreted by *Sphingomonas elodea*. Native Ge is partially esterified with acyl substituents of l-glycerate and acetate at the C-2 and C-6 positions of the (1-3)-linked d-glucose. Acetyl groups are usually removed by alkaline treatment, leading to deacetylated Ge, which is capable of forming physical cation-induced hydrogels; this property makes Ge suitable for the development of in situ gelling controlled release systems. Indeed, in the last years, several studies demonstrated the potential use of Ge in tissue engineering [12]. Moreover, it has been shown that Ge can be suitable for the formulation of nano-scale systems: in aqueous environment, hydrophobic modifications of the polymer chains with small molecules (e.g., cholesterol or prednisolone) lead to the formation of self-assembled nano-structures that are suitable for drug delivery purposes [13,14,15].

Starting from these key features, cholesterol-modified gellan gum (GeCH) was thus exploited for the formation of a new class of gels. In particular, the present work aimed to design a novel depot system based on close-packed liposomes combined with the cholesterol-modified gellan gum. Close-packed vesicles can be obtained by using a high concentration of phospholipids, and these systems showed a great ability in diclofenac skin delivery [16]. Vesicles were obtained using high concentrations (120–300 mg/mL) of a commercial mixture of soy lecithin, Phospholipon 90G (P90G), and they were simultaneously prepared and gelled with the cholesterol-modified gellan, thus forming a networked system formed by vesicles and polymer chains. The size and polydispersity of the vesicles’ dimensions and the viscoelastic behaviour of the resulting gel systems were determined. Finally, the thus-formed hybrid gels were studied for their release properties of the anti-inflammatory agent, diclofenac, for biomedical purposes.

## 2. Results and Discussion

The idea underlying the present work is the possibility of forming bridges mediated by the GeCH chains among the liposomes suspended in an aqueous environment, thus forming a hydrogel network. The cholesterol pendant groups, chemically linked to the polymer chains, ensure the “anchorages” of the polymer chains to the liposomes, leading to a tri-dimensional physical network capable of entrapping water. It is worth noticing that even though GeCH is insoluble in aqueous media [13,15], it can be easily dispersed in water thanks to the amphiphilic nature of the polymer chains. The resulting network based on liposomes and polymer chains can thus be referred to as a hydrogel system, which, due to its complex structure, can be defined as “hybrid gel”.

Because of its soft nature and the possibility of preparing liposomes and GeCH suspensions separately, these hybrid gels can be exploited in the biomedical field for in situ gel depot applications.

To reach the above-stated end-point, we studied the interaction between liposomes and GeCH in order to define the appropriate combination of the two systems, leading to the formation of a soft hybrid gel. For this purpose, hybrid systems based on P90G and GeCH were prepared, mixing the components at various concentrations.

### 2.1. Tube Inversion Tests

Table 1 reports the macroscopic behavior in terms of the physical state of the different mixtures prepared at several P90G and GeCH concentrations. Note that the “gel state” in these experiments has been defined operatively as the “no flowing condition” of the system under its own weight (gravity) in an inverted tube test. The systems at the lowest P90G concentration (120 mg/g) and GeCH at all the tested concentrations (up to 10 mg/mL) remained liquid, whereas at higher P90G concentrations (180–300 mg/g), a soft solid hybrid gel was obtained. According to the model depicted above, this behavior could be ascribed to the establishment of stronger forces between the polymer chains and liposomes in the latter conditions.

### 2.2. Particle Size Measurements

In the proposed model, the cholesterol-mediated interactions between the polymer chains and the liposomes should certainly affect the dimensions of the vesicles, as the interaction of the cholesterol moiety with the outer shell of the vesicles modifies the interactions among the phospholipids in the bilayer [7,8]. At the same time, the polymer chains—acting as bridges among the vesicles, and interacting with the surface of the vesicles—influence both the dielectric constant of the solvent and the interfacial tension of the vesicle, thus influencing the vesicle dimensions.

In Table 2, the size (d) and polydispersity index (PDI) of the liposomes at the tested concentrations (120–300 mg/g) are reported. The dimensions of the vesicles ranged between 140 and 170 nm, depending on the phospholipid concentration.

**Figure 1:**
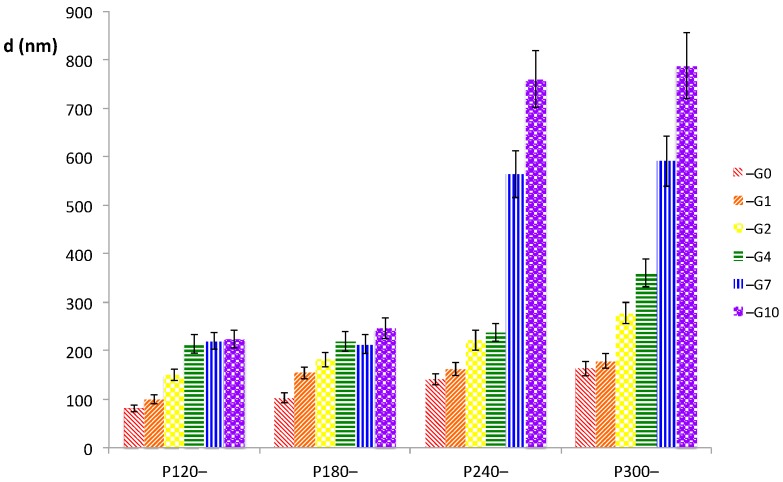
Particle size (d) of the different hybrid gels measured with DLS. Results are reported as mean value (*n* = 3).

The size of the liposomes interacting with GeCH in the various prepared mixtures are reported in Figure 1. Particle sizes were strictly related to both the P90G and the GeCH concentrations; indeed, the sizes strongly increased as the concentrations of the two mixed systems were increased. This trend was particularly evident at the highest concentrations of both liposomes and GeCH (P90G 240–300 mg/g, GeCH 7–10 mg/mL). A correlation between particle size and the physical state of the mixture reported in Table 1 can be easily assessed.

### 2.3. Rheological Characterization

Rheological characterization is a useful tool to define the suitability of systems for specific application; the obtained information can also be exploited to confirm—from a macroscopic point of view—a model provided at a molecular level. In the present study, the rheological characterization of the samples (i.e., the initial solutions and the hybrid gels obtained using the highest P90G concentrations of 240 and 300 mg/g) was carried out.

**Figure 2:**
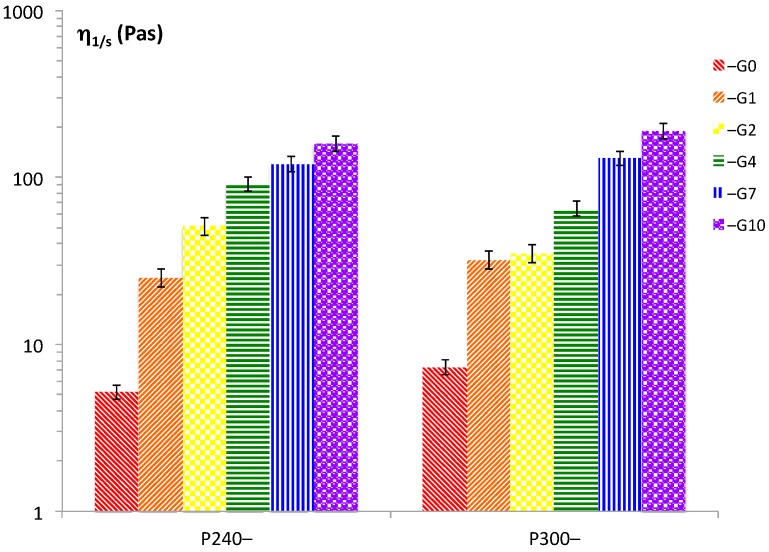
Viscosities of P90G/GeCH mixtures, obtained at 25 °C, 1 s^−1^, for the P90G and GeCH systems, at various concentrations (240–300 mg/g and 0–10 mg/mL, respectively). Results are reported as the mean value (*n* = 3).

The shear flow experiments evidenced the viscosity profiles of the various systems; the values of the viscosities measured at 1 s^−1^ are reported in Figure 2. Upon increasing the concentrations of P90G and GeCH, the viscosities of the resulting gels become higher. However, the observed increase in viscosity could not be due only to a concentration effect (water coordination) or to a simple additive effect of the viscosities of the two systems. Indeed, mixing GeCH with P90G led to more than an additive increase in viscosities. Note that the GeCH suspensions at 1, 2, 4, 7, and 10 mg/mL showed values of 0.003, 0.005, 0.01, 1.32, and 12 Pa⋅s at 1 s^−1^, respectively. Therefore, looking at the values reported in Figure 1 and Figure 2, inter-particle interactions and modification of the liposome dimensions had an important effect in the P90G/GeCH viscosities, thus confirming the proposed model.

Rheological experiments in oscillatory shear experiments were carried out to determine the elastic and loss moduli (G’ and G’’ respectively) of the prepared systems. The resulting phase lags (tan δ = G’’/G’) were also determined. 

**Figure 3:**
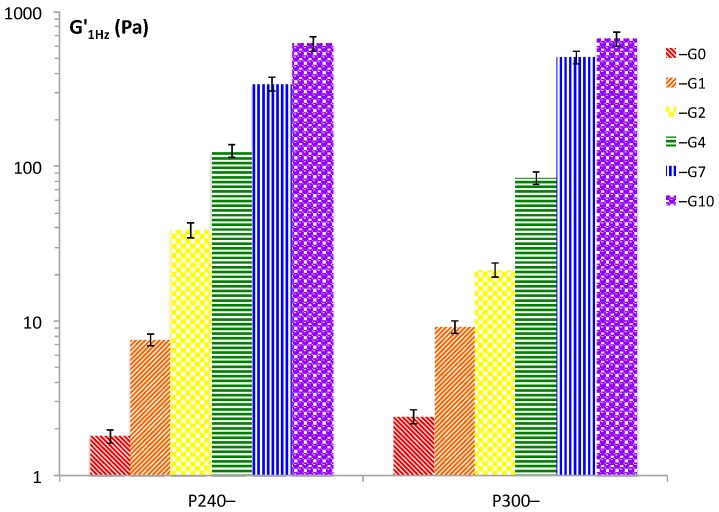
Storage Modulus, G’, obtained by means of frequency sweep experiments (25 °C, 1 Hz) for the different hybrid gels. The data are reported as the mean value (*n* = 3).

**Figure 4:**
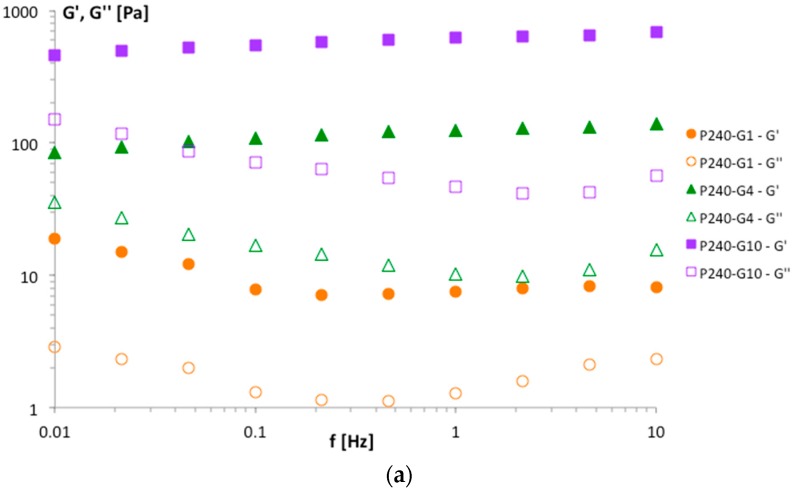

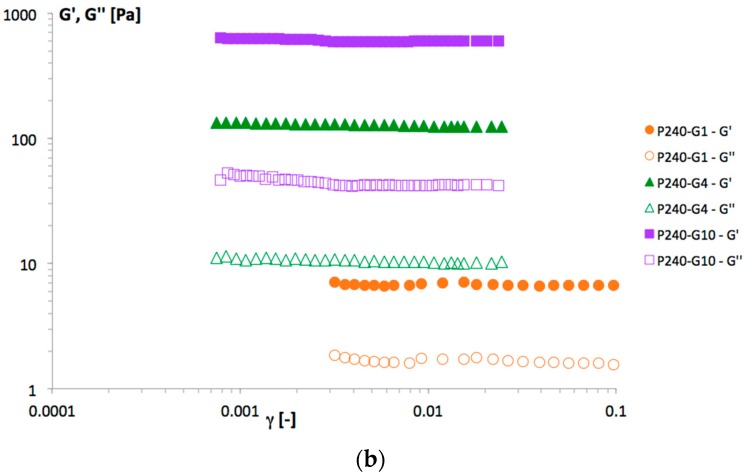
(**a**) Representative mechanical spectra (*T* = 25 °C) and (**b**) strain sweep experiments (*T* = 25 °C) for hybrid gels prepared with different GeCH concentrations: P240-G1 (in orange), P240-G4 (in green), and P240-G10 (in purple). G’: full symbols; G”: empty symbols. The tests were performed in triplicate, and standard deviations always laid within 10% of the mean.

The values of the storage moduli, G’, of the P90G/GeCH mixtures (obtained at 1 Hz) reported in Figure 3 evidenced a trend in agreement with the viscosity data: increasing P90G and GeCH concentrations led to the formation of more rigid hybrid gels. Representative frequency sweep spectra of the samples P240-G1, P240-G4 and P240-G10 are reported in Figure 4. For these samples, a decrease in the phase lag values from 0.17, 0.08, and 0.07 for P240-G1, P240-G4, and P240-G10, respectively, was observed. This means that increasing amounts of GeCH led to the formation of more elastic materials; this effect could be ascribed to the formation of an increasing amount of cross-linking in the network due to an increase of the overall inter-particle interactions.

### 2.4. Water Uptake Experiments

The ability of these hybrid gels—P240-G7, P240-G10, P300-G7, and P300-G10—to absorb water was investigated, and the data are reported in Table 3. This property is an important feature in terms of the suitability of these hybrid gels for depot applications. The in vitro experiments carried out in the present study should be intended as a model description of the behavior of these gels, as the in vivo conditions are dependent on the application sites.

With this warning in mind, when a low amount of water is used (0.5 mL water for 1 g of gel), the water uptake, Q, was independent of the gel composition: all the different gels absorbed all of water used in the experiment. When a higher amount of water was used (1 mL for 1 g of gel), it was observed that the Q values depended on the gel composition: the samples at the highest concentrations (P300-G7 and P300-G10) showed the higher water uptake. This effect can be explained in terms of osmotic pressure of the gel; as a general rule, the higher the mass and particle concentration, the higher the internal pressure of the gel [17]. At higher amounts of added water (i.e., gel:water ratio 1:2), the erosion of the gels starts to become significant and loss of mass should be taken into account. 

### 2.5. Diclofenac Release

The ability of the P90G/GeCH hybrid gels to ensure a sustained release of the model drug diclofenac (DCF) was studied as a function of time in sink conditions. The choice of DCF was due to the interest in releasing “locally” anti-inflammatory drugs using depot systems, avoiding systemic administration, thus reducing side effects. Hybrid gels at higher mass concentration were put into dialysis tubes in water at 37 °C, and the release of DCF from the hybrid gels was followed for 8 days. As a control, the release of free DCF at the same concentration and experimental conditions was also investigated. 

In the experiments, DCF was loaded within liposomes without any further purification; therefore, at the end of the preparation, the liposome suspensions contained both DCF embedded in the vesicles and DCF in the surrounding water medium. Stemming from the interaction with GeCH, the hybrid gel samples thus presented DCF in two different forms: the first one, embedded within the liposomes, and the second one, dispersed in the environment around the liposomes (i.e., the gel state). This choice allowed the comparison of the different systems, normalizing for the overall drug concentration. In these conditions, our goal was to demonstrate the feasibility of a depot system by using a drug delivery device, where a fraction of the drug can be released by diffusion (drug in the inter-vesicle environment), whereas a second amount of the drug is embedded in a reservoir (within the liposomes).

**Figure 5:**
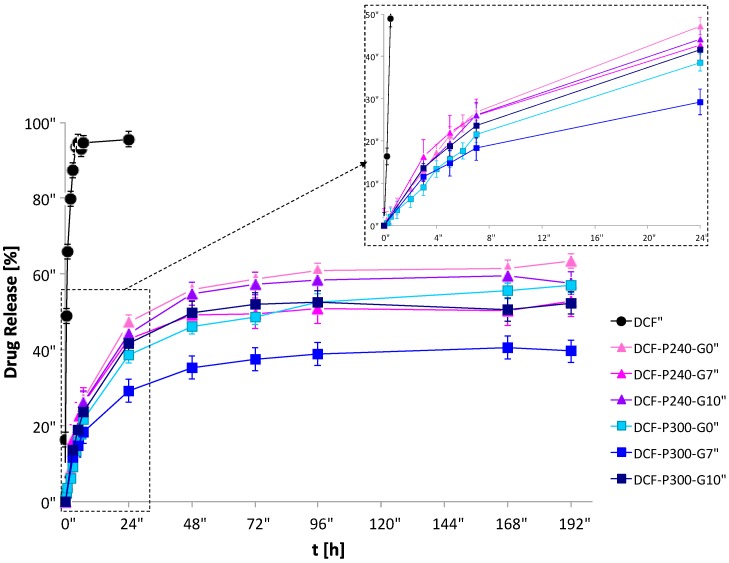
Release profiles of free DCF, DCF-loaded within the liposomes and DCF-loaded within the hybrid gels. Results were obtained through dialysis in water at 37 °C. Curves represent the release of DCF from: water solution, as control (black), P240 hybrid gels (P240-G0, P240-G7, and P240-G10 light pink, pink, and purple, respectively), and P300 hybrid gels (P300-G0, P300-G7, and P300-G10 light blue, blue, and dark blue, respectively).

Obtained data are reported in Figure 5. Free DCF (control) was totally released in few hours, whereas part of the DCF in the hybrid gels was still retained even after 8 days. Since 40–70% of loaded DCF was not released from the hybrid gels over 8 days, P90G/GeCH systems were solubilized in H_2_O/EtOH, and the amount of loaded DCF into the hydrogel matrix was confirmed after that period of time. The percentage of not released drug, spanning in the range 40–70%, depended on the gel nature and the entrapment efficiency of liposomes. Moreover, as expected, release rates from hybrid gels were slower than those of free liposomes, confirming the ability of the GeCH matrix to improve the controlled release of DCF.

The obtained results clearly indicate that the releases of DCF from the hybrid gels were modulated in a complex way. As a general observation, the higher the concentration of P90 and GeCH, the lower the amount of DCF released. 

## 3. Conclusions

In the present work, close-packed liposomes based on high concentrations of the phospholipid P90G and the water-insoluble cholesterol derivative of the polysaccharide gellan were combined in order to form hybrid gels. These gels could be obtained by mixing the two components at high concentrations. The proposed model of the resulting network can be depicted as a dispersion of liposomes networked by the polymer chains, previously interacting with the liposomes’ surfaces by cholesterol pendant groups.

According to this model, liposomes’ dimensions increased as the concentration of GeCH was increased. Moreover, the rheological characterization in shear conditions confirmed the interaction between liposomes and polymer chains, as their combination resulted in “not flowing” systems, showing mechanical spectra typical of gels and viscosities values more than additive. In terms of elasticity and viscosities, the mechanical properties are suitable for biomedical applications of the gel, and the overall characteristics indicate that these gel systems can be exploited in depot applications. Indeed, the two components can be combined locally, immediately stemming the gel. To study the properties of these hybrid gels as drug delivery devices, the capability of these systems to be loaded with, to carry, and to deliver DCF in aqueous environment were investigated. This anti-inflammatory drug was chosen as model drug for local therapy applications, as well as long lasting systemic release. Drug delivery experiments confirmed the suitability of these hybrid gels as drug carrier as well as long lasting drug release devices. The in vitro experiments described in the present work confirmed the possibility of modulating the release of the drug, even in sink conditions, for at least eight days. More importantly, part of the drug remained embedded in the gels, within the liposomes, acting as a reservoir system that can be exploited in in vivo conditions for long-lasting delivery applications.

The hybrid gels systems based on P90G and GeCH can thus represent an important tool to be exploited in drug depot applications.

## 4. Materials and Methods

### 4.1. Materials

Gellan gum sodium salt (Ge-Na) Kelcogel® CG-LA was purchased from Giusto Faravelli (Milano, Italy), soy phosphatidylcholine Phospholipon® 90G (P90G) was purchased from Lipoid GmbH (Ludwigshafen, Germany), cholesterol (CH) was purchased from Carlo Erba Reagents (Italy), diclofenac sodium salt (DCF) was purchased from Sigma Aldrich (Cornaredo, Italy). All other reagents were purchased from Sigma Aldrich (St. Louis, MI, USA). All products were used without further purification. 

### 4.2. Synthesis of Gellan–Cholesterol

Gellan–cholesterol synthesis was carried out, modifying the procedure described in previous works [13,15]. Cholesterol was derivatized with 4-bromo-butyric acid, leading to the Br-butyric-cholesterol (CH-Br) derivative. Cholesterol (500 mg, 1.3 mmol) and Dimethylaminopyridine (DMAP) (79 mg, 0.65 mmol) were solubilized in CH_2_Cl_2_ (7 mL), and the solution was stirred for 30 min. Separately, *N*-(3-Dimethylaminopropyl)-*N*′-ethylcarbodiimide hydrochloride (EDC·HCl) (744 mg, 3.9 mmol) and 4-bromobutyric acid (648 mg, 3.9 mmol) were solubilized in CH_2_Cl_2_ (7 mL), and the solution was stirred for 30 min. The two solutions were then mixed, and the reaction was kept overnight at room temperature and monitored by thin layer chromatography (TLC) on silica gel (eluent ethylacetate:cyclohexane 1.5:98.5). The solution was then washed with NaOH (0.05 M), HCl (0.05 M), and distilled water (three times); finally, reaction product was dried with anhydrous Na_2_SO_4_. Solvent was removed with rotary evaporator, and the crude product was purified on silica column (ethylacetate:cyclohexane 15:85). The yield of reaction was 70% (by weight). The Br-butyric-cholesterol (Br-CH) product was analyzed by ^1^H-NMR (400 MHz) spectroscopy [13]. 

Native gellan gum Na salt was first converted into tetrabutylammonium (TBA) salt form (Ge-TBA) with an ion-exchange resin (Dowex 50 × 8), and then depolymerized using a microfluidizer (M-110EH—Microfluidics Corp, Newton, MA, USA, 87 μm chamber G10Z, inlet pressure 1200 bar, 7 passes). 

Then, GeCH was synthesized by dissolving the obtained low Mw Ge-TBA (1 g, 1.125 mmol of repeating units) in 80 mL of *N*-Methyl-2-pyrrolidone (NMP) and by adding Br-CH (120 mg, 0.225 mmol), previously dissolved in NMP (15 mL); the reaction was kept under magnetic stirring for 40 h at 38 °C. Dialysis against distilled water (Visking tubing, cut-off: 12,000–14,000) was carried out until constant conductivity was reached; GeCH was finally freeze-dried and recovered as white powder. 

### 4.3. Hybrid Gels Preparation

#### 4.3.1. Preparation of GeCH suspensions

GeCH was dispersed in bidistilled water for 2 h at 75 °C at different concentrations (1, 2, 4, 7, and 10 mg/mL). 

#### 4.3.2. Preparation of Liposomes and DCF-Loaded Liposomes

P90G were swollen at concentrations: 120, 180, 240, and 300 mg/g in bi-distilled water at 50 °C, overnight. Dispersions were then vortexed for 10’ and sonicated with a probe-type sonicator (Vibra Cell—VC 750, microtip 6.5 mm) for 150 s (sonication conditions: 20 kHz, 30% of amplitude, pulsed mode: 5 s of sound–2 s of silence).

To prepare drug-loaded hybrid gels, the same procedure was performed, but a proper amount of DCF was added to the P90G/water mixture in order to obtain a final drug concentration of 20 mg/g of liposomes suspension. The suspensions of DCF/liposomes were used without purification; i.e., the free DCF not embedded in liposomes was not removed from the suspension.

#### 4.3.3. Hybrid Gels Preparation

**Scheme 1:**
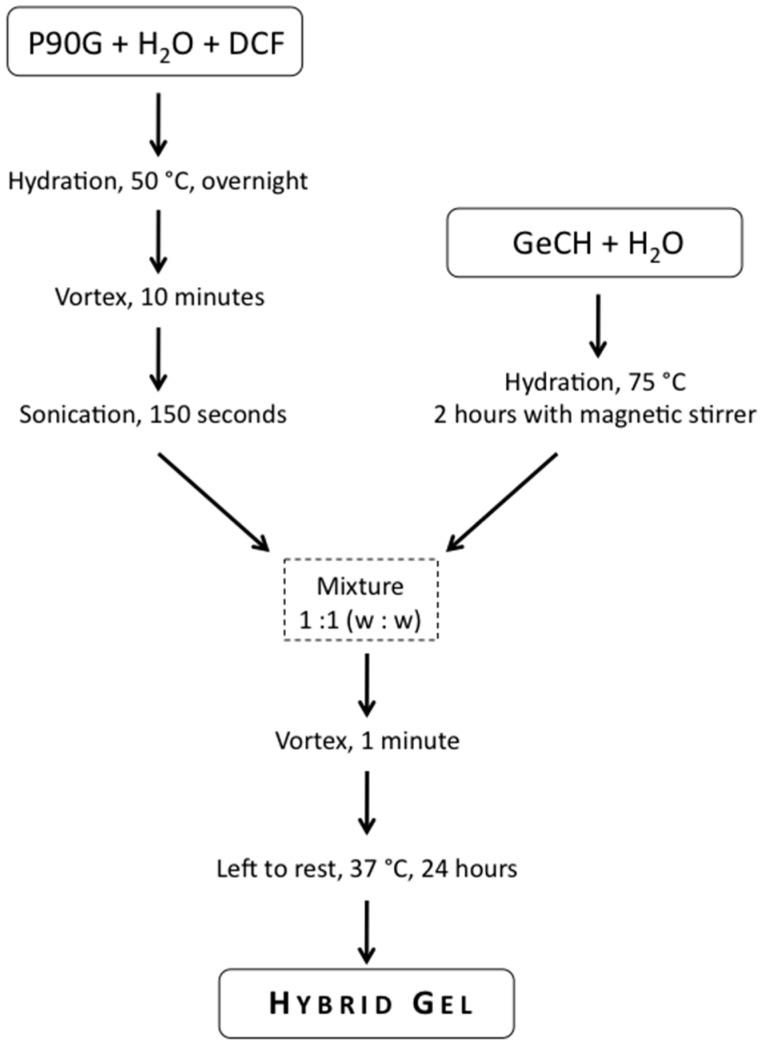
Schematic description of the procedure for the hybrid gels preparation.

The preparation of the hybrid gels is summarized in Scheme 1. The hybrid gels were prepared by adding 1 g of GeCH suspension of the prepared suspension (as above described; concentrations in the range 1–10 mg/mL) to 1 g of liposomes suspension (120–300 mg/g). Mixtures were then vortexed for 1 min until a homogenous distribution was obtained, and left at 37 °C for 24 h. The evaluation of the gel formation was performed using the tube inversion test. To prepare blank samples for all experiments, liposome suspensions were mixed with pure water (GeCH 0 mg/mL). In Table 4, all the hybrid systems prepared are reported. DCF-loaded hybrid gels were prepared following the same procedure (DCF-loaded liposomes were used): the DCF final concentration in the loaded hybrid gels was 10 mg/g. 

### 4.4. Measurement of Particle Size

The size and polydispersity index (PDI) of the samples (liposomes suspensions and hybrid gels) were determined by means of dynamic light scattering (DLS) using a Submicron Particle Sizer Autodilute Model 370 (Nicomp, Goleta, CA, USA). Samples were diluted before the measurement to reduce light intensity absorption. 

### 4.5. Rheological Characterization

Rheological experiments were carried out in shear regime with a controlled stress Haake RheoStress 300 Rotational Rheometer (Thermo Fisher Scientific, Waltham, MA, USA), equipped with a Haake DC10 thermostat. The geometry used for the tests was a cone–plate geometry (Haake CP60Ti: diameter = 60 mm; cone = 1°; gap = 0.053 mm). Mechanical spectra (frequency sweep experiments) were recorded at 25 °C in the range 0.01–10 Hz, in the linear viscoelastic region, applying a constant deformation (γ = 0.01), preliminarily assessed by stress sweep experiments. Stress sweep experiments were performed at 25 °C and 1 Hz in the range 0.01–30 Pa. Flow curves of the solutions were determined at 25 °C using the above reported geometry in the range 0.001–1000 s^−1^. A stepwise increase of the stress was applied, with an equilibration time of 30 s. All measurements were performed in triplicate. 

### 4.6. Water Uptake Experiments

The water uptake capability of the hybrid gels was determined gravimetrically. All hybrid gels (about 1 g) were placed in the bottom of Eppendorf tubes and accurately weighted (*w*_0_). The tubes were then centrifuged (5 min, 3000 rpm) to obtain pellets firmly attached at the bottom of the tubes. Water (0.5, 1, and 2 mL, respectively) was then added to the samples, and the samples were stored at 37 °C; after 6 hours, the supernatant was removed, and samples were weighted (*w*_t_). The water uptake value, Q, was evaluated by (*w*_t_ − *w*_0_)/*w*_0_. All measurements were performed in triplicate.

### 4.7. Drug Release Experiments

A weighted amount of DCF-loaded hybrid gel (about 1 g) was put in a dialysis tube (regenerated cellulose, MWCO 12,000–14,000); both ends were tied, and the final tube length was about 5 cm. The tube was suspended in 35 mL of water at 37 °C. The dissolution medium was magnetically stirred at 200 rpm. At defined time points, 3 mL were taken off and replaced with 3 mL of fresh medium. The released DCF was determined at λ 276 nm using a Perkin-Elmer, lambda 25, UV-vis spectrophotometer (PerkinElmer, Waltham, MA, USA) with quartz cell (path-length 1.0 cm). Calibration curve of DCF in water was built in the range concentration of 2–25 μg/mL.

After 8 days, hybrid gels still entrapped into the dialysis tubes were recovered, diluted with H_2_O/EtOH 10/90 (*v*/*v*) to dissolve liposomes, and centrifuged (4000 rpm, 15 min). The supernatant was analyzed in the UV-vis region and the absorbance at λ = 284 nm was registered in order to determine the amount of unreleased DCF; calibration curve of DCF in H_2_O/EtOH 10/90 (*v*/*v*) was built in the range concentration of 2–25 μg/mL. 

For comparison, the release profiles of free DCF (10 mg/mL) and DCF loaded within liposomes were also registered. All the measurements were performed in triplicate, and standard deviations were reported.

## Figures and Tables

**Table 1:** Physical state of Phospholipon 90G/cholesterol-modified gellan gum (P90G/GeCH) systems prepared at various concentrations. Results were obtained from the tube inversion test. “L”: fluid behaviour; “G”: soft solid.

GeCH Stock Suspension	P90G Stock Suspension (mg/g)			
	120	180	240	300
0 mg/mL	L	L	L	L
1 mg/mL	L	L	L	L
2 mg/mL	L	L	G	G
4 mg/mL	L	L	G	G
7 mg/mL	L	G	G	G
10 mg/mL	L	G	G	G

**Table 2:** Size (nm) and PDI of P90G stock dispersions determined by dynamic light scattering (DLS). PDI: polydispersity index.

P90G	d (nm)	PDI
120 mg/g	141 ± 12	0.37 ± 0.05
180 mg/g	151 ± 18	0.34 ± 0.08
240 mg/g	166 ± 22	0.34 ± 0.04
300 mg/g	172 ± 19	0.40 ± 0.11

**Table 3:** Water Uptake values, Q, for hybrid gels obtained in different experimental conditions at T = 37 °C: gel/water ratio = 1:0.5, 1:1 and 1:2, respectively.

Sample	Q		
	Gel/Water 1:0.5	Gel/Water 1:1	Gel/Water 1:2
P240-G7	48%	15%	17%
P240-G10	49%	25%	50%
P300-G7	49%	48%	47%
P300-G10	49%	48%	33%

**Table 4:** Composition and nomenclature of the different hybrid gels obtained. The corresponding diclofenac-loaded samples are identified as: DCF-Px-Gy.

GeCH Stock Suspension	P90G Stock Suspension (mg/g)			
	120	180	240	300
0 mg/mL	P120-G0	P180-G0	P240-G0	P300-G0
1 mg/mL	P120-G1	P180-G1	P240-G1	P300-G1
2 mg/mL	P120-G2	P180-G2	P240-G2	P300-G2
4 mg/mL	P120-G4	P180-G4	P240-G4	P300-G4
7 mg/mL	P120-G7	P180-G7	P240-G7	P300-G7
10 mg/mL	P120-G10	P180-G10	P240-G10	P300-G10
